# Cases of Cancer Uteri

**Published:** 1831-01-01

**Authors:** 


### CLINICAL REVIEW.

### LXVII.

### SIR PATRICK DUN'S HOSPITAL.

CASES OF CANCER UTERI. By W. F. MONTGOMERY, A.M. &c. Professor of Midwifery to the King and Queen's College of Physicians in Ireland, &c.*

ON the importance of being well acquainted with diseases that at present are not curable by medicine, as well as with those that are so, we need say nothing here. If only for diagnosis' sake, they are subjects of anxious and careful study to the scientific practitioner of medicine. Diseases of the uterus are extremely common, and the malignant affections are not unfrequent, but again and again are women pronounced to labour under them, when they do not

* Dublin Hospital Reports, Vol. V.

in reality exist. Dr. Montgomery, the author of the paper we are now to notice, has witnessed eighteen cases of cancer uteri since the commencement of the year before last, and he laudably communicates to the public what he thinks is worth its consideration. At the same time, he regrets that he must disclaim altogether being able to point out any new means of giving relief. This is modest, and what we might expect from a man of considerable experience. A person who had seen but two or three cases in his life, would probably have written to the praise and glory of some almost untried specific.

*Case* 1. "On the 15th, Aug. 1827, I was called on to examine I. Henry, in Sir Patrick Dun's Hospital. She stated her age to be 41, but appeared older; she had been five months ill, and now complained of severe lancinating pains in the small of the back, and darting 'like lances' in different directions through the pelvis, particularly in the left iliac region, in the direction of the sigmoid flexure of the colon, and commencement of the rectum, in which situation she described it as a burning pain, and this sensation of burning was also severely felt about the internal margin of the anus; the lancinating pain extended also down the inside of the thighs, along the course of the obturator nerve.

There was a copious sanious discharge of a fetid odour from the vagina, and on making an examination, I found the os and cervix uteri enlarged, hard, and so low in the pelvis as not to be more than two inches from the external parts: the labia of the os uteri being both enlarged and projecting, made the orifice feel to the touch open and gaping; the surface with which the finger came in contact was very unequal, and communicated the sensation that would be experienced by passing the tip of one's finger over and among the tops of all the fingers of a hand collected together; the upper part of the vagina and uterus were immoveable, as if firmly united to the adjacent structures."

This state of the uterus soon began to alter; early in October ulceration began, and at length it had proceeded so far, that it was difficult to feel any distinct boundary of the uterus. On the 23d Nov. the legs were found slightly anasarcous, and the patient said that she had passed no water for three days, and that she felt no desire to void any. Two days afterwards the anasarca had spread, and still no urine had been passed. The catheter was introduced, but none escaped. This went on till the 27th, when the anasarca had become extreme, and suddenly a gush of water, like urine, came from the vagina, and continued to flow without intermission for 48 hours, the anasarca so rapidly decreasing, that on the 30th there was hardly any of it left, nor did it again recur. Incontinence of urine remained, and, on examination, the posterior part of the cervix of the bladder was found to have sloughed away, leaving an aperture into the bladder from the vagina, through which the thumb would readily pass. In addition to the usual distressing symptoms, violent vomiting in a few days took place, and although stopped, for a brief interval, by the application of folds of lint, soaked in the acetum opii, to the epigastrium, it proceeded, and carried off the patient on the 24th December.

*Sectio Cadaveris.* The great omentum descended into the pelvis, and was adherent all over the fundus uteri, and uterine extremities of the fallopian tubes. The ureters were the size of the large intestine of an infant, their coats were quite transparent, and their length was so increased that they were quite serpentine in their course. On dissecting out the pelvic viscera, and making a longitudinal section of the rectum from behind, there was seen, about eight inches from the anus, a stricture of the gut, reducing its diameter to scarcely half an inch. The intestine, for about two inches above this stricture, and four or five inches below it, presented on its internal surface the true scirrhous deposit, and its coats, especially about the stricture, were greatly thickened; the portion next the anus was healthy, with the exception of a few hæmorrhoidal tumours. All the cervix, and great part of the body of the uterus, were gone, and the remainder was double its natural size and thickness. The posterior surface of the bladder, comprising the cervix and entrance of the ureters, had been destroyed, leaving an opening equal in diameter to a half-crown-piece. The internal surface of the bladder exhibited the same morbid alteration as the rectum.

"The pathological changes here observed account in a very satisfactory manner for some part at least of the symptoms, the cause of which during life was, at best, but a matter of conjecture, I allude to the suppression, and afterwards incontinence of urine, and their relation to the anasarca.

It seems to me almost demonstrable, that the cause of the suppression of urine was the mechanical closure of the orifices of the ureters by the thickening of the parts surrounding them, which took place in consequence of the inflammation (which I believe to be always the first stage of carcinomatous affections) having seized on that part of the bladder; so that the urine could not effect its entrance into that organ, while at the same time it was rapidly secreted by the healthy kidneys, and poured into the ureters until they became greatly distended, and then another process was set up in the system to compensate for the usual urinary excretion, and the superfluous fluids were poured into the cellular membrane in different parts of the body, from whence they were again as rapidly recalled as soon as a vent was re-established for their discharge, which we know was effected by the death and separation of that portion of the bladder into which the terminations of the ureters penetrate, in consequence of which, incontinence took place, and a perpetual draining of fluid from the system was established, by which, as already mentioned, the anasarcous effusion was completely removed at the end of two days.

A change in the state of the urethra took place in this patient, which I have almost always observed to happen when the bladder becomes involved in the disease, and particularly when urine is discharged from it without passing through the urethra. I allude to a remarkable contraction of the external extremity of that duct, and of its orifice in particular, so that even a small catheter or bougie cannot be introduced without considerable difficulty, and of course the infliction of much pain; in consequence of this, if an attempt be made to introduce the same catheter which we may have used with the patient, previously with the utmost facility, it will be found quite impracticable to pass it without causing great distress and pain; aware of the probable existence of this constriction, we should therefore in such cases, I think, always use an instrument of a small size."

*Case* 2. On the 9th Feb. 1828, Dr. Montgomery was called to see Ann Clark, æt. 52, who had had eleven children, the youngest nine years old, and miscarried once. In Dec. 1827, she first perceived a swelling of the left thigh, attended with some pain and soreness about the groin. The whole limb was now considerably swollen and anasarcous, and some of the inguinal glands were enlarged, hard, and tender to the touch. She complained of some pain along the limb, of sharp pains in the lower part of the abdomen, darting downwards, through the pelvis, towards the uterus, and there was some leucorrhœal discharge from the vagina. On examination, the os and cervix uteri were enlarged, indurated, and very irregular, and the parts about the upper end of the vagina consolidated into one mass, rendering the uterus quite immoveable. The functions of the bladder and rectum were not interfered with; the appetite was tolerably good. Iodine was tried, but disagreed, and was omitted. All at once, the discharge from the vagina changed its character to pus, mingled with a brownish matter and small shreds, and then the usual melancholy train of symptoms set in. Some difficulty in passing the stools occurring, the rectum was found to be compressed, about four inches from the anus, by the enlarged and scirrhous glands in the pelvis. The effect of this was obviated by keeping the bowels open. On the 29th July the patient died. There had been no hæmorrhage from the vagina.

"Extreme emaciation of the whole body: the pelvic glands were so enlarged that the cavity of the pelvis was completely filled by them, and the uterus quite borne up out of its natural situation; the rectum also from the same cause, was pushed out of its usual course and descended along the right side of the sacrum instead of the left, and at about four inches from the anus its calibre was so diminished that it would not receive more than the little finger into its cavity; this portion was completely girt about by the enlarged pelvic glands, and when cut open the coats of the intestine were observed to be considerably thickened at this spot: this at once explained the cause of the complaint made by the patient, 'that a bone had got into the gut:' in other respects the intestine was healthy.*

The uterus was very extensively destroyed by the ulceration, as was also the upper portion of the vagina; the cervix uteri and the lower half of the body were altogether gone; the upper part of the body and fundus were reduced to one half of their natural thickness by ulceration on their internal surface, in consequence of which the upper part of the organ was rendered quite hollow, and would easily contain a fig.

The bladder had become engaged in the disease; a portion of it, equal in extent to the size of a crown piece, at the back part and toward the right side, had become affected with true scirrhous thickening, the coats being about half an inch thick, and of almost cartilaginous hardness; but no ulceration had taken place, nor was there any dilatation of the ureters.

In the integuments of the left side, between the margin of the ribs and the crest of the ileum, there was a small round tumor of about an inch in diameter, which on being cut presented the scirrhous structure; and there were in the omentum some smaller tumors of a similar character. The enlarged pelvic glands, in this case, and I had occasion to make the same remark in several other instances, presented very different characters, some being as hard as cartilage, and uniform in their texture; others of a caseous consistence; while others presented the scirrhous character so perfectly, that I have preserved a section of one of them, as a specimen of that morbid structure."

*Case* 3. Sarah Kearney, æt. 36, came under Dr. Montgomery's care on the 12th June, 1828. She had severe pain in the back, darting through the pelvis and down the inside of the thighs, and a discoloured and offensive discharge from the vagina. On examination the os uteri was found low in the vagina, enlarged, hardened, and irregular. The upper extremity of the vagina was consolidated with the surrounding parts, the recto-vaginal septum unusually thick, and firm, but there was no inconvenience in discharging either the bowels or bladder. She had been three months ill, and the complaint had commenced with irregularity in the catamenia, followed by uterine hæmorrhage, of which she had had repeated and very profuse returns both before and since her ad-

* "In the Ed. Med. Journ., Jan., 1829, p. 220, a case is recorded in which constipation was induced by this kind of compression, and lasted *nine weeks;* 'all efforts to procure the passage of the fæces, either by injections thrown up in great quantity or by bougies, completely failed.'

'During this time the patient was subject to occasional paroxysms of severe pain in the bowels, accompanied with most distressing sense of distention; *but her sufferings were by no means permanent:* and except upon the occurrence of such fits she felt *no very great uneasiness*, and was even able to get out of bed and exert herself in her usual occupations. Her pulse was little disturbed; her appetite tolerably good; and her strength not very materially impaired: no vomiting of fœcal matter; indeed no vomiting of any kind occurred during the continuance of the disease."

mission into the hospital. On the 24th July she began the use of the tinct. iodinæ, and in 10 days a marked amendment had taken place. The quantity of the iodine was gradually increased from seven drops to fifteen three times daily. The general symptoms were vastly improved, and on the 25th Aug. the uterus was found decidedly reduced in size, much softer, and more mobile. She went on till the 19th Sept. when she seemed restored to perfect health, but on the 20th, tremors and the other bad symptoms produced by iodine set in, and it was necessarily discontinued. Early in October all the former unfavourable symptoms were renewed, and iodine only brought a partial relief. At one time she had many of the symptoms of organic disease of the heart, which were removed by antispasmodics. In May, 1829, perforation of the bladder took place, the patient went into the country, and there she died. She was not examined after death.

"The perforation of the bladder was preceded in this patient by a train of symptoms, which although much less marked than in the case of Henry, are I think worth noticing, because I have observed them to occur very generally in such cases, announcing the approach of that miserable change: about five, six, or seven days before the slough takes place the patient tells you she is troubled with the gravel and has great difficulty in passing water, which she accomplishes with pain; next she tells you that she passes much less than usual; and lastly, for a day or two before the incontinence commences, she passes hardly a drop, perhaps none, and then all at once it flows involuntarily. In such cases, after death, I have always found the ureters greatly dilated. —See p. 417. In those cases where considerable disturbance in the functions of the bladder exists perhaps from the commencement, these premonitory symptoms cannot, of course, be distinguished.

The iodine in this case appeared to restrain uterine hemorrhage, not to excite it, an effect generally apprehended from its use.

This may be considered as an early instance of the disease."

*Case* 4. Esther Henright, æt. 30, seen May 7, 1829. Has had five children; was married at the age of 14; before 15 was affected with syphilis; and has led a very irregular life. Four months ago she fell in the street and struck her hip and side. Immediately afterwards she had a profuse hæmorrhage from the vagina, which has frequently returned since. She had little discharge until two or three weeks ago, when she began to suffer pain and to have a constant discharge, which is generally discoloured. She had not the aspect of organic disease. On examination the os uteri was found low in the vagina, enlarged, hard, irregular, quite consolidated with the top of the vagina to the neighbouring parts. On applying the speculum there were distinctly seen patches of ulceration which had partially destroyed the os uteri and extended to a distance of more than half an inch within the cervix. The disease marched on as usual—in July perforation of the bladder took place—the patient suffered from very severe pain shooting from the centre of the pelvis through the right hip, and in the course of the sciatic nerve, and best relieved by the hip-bath, with a stimulating anodyne liniment—during the last five or six weeks of life there was incessant vomiting, accompanied with slight pain, and tenderness on pressure over the stomach, but not in the other parts of the abdomen. She died on the 24th Sept.

*Sectio Cadaveris*. Universal peritoneal inflammation—small intestines adherent, in three or four places, to the fundus uteri, as was the great omentum—cervix and great part of the body of the uterus destroyed, as was a portion of the bladder as large a half-crown—ureters distended to the size of a swan's quill.

This was one of the earliest instances of the disease, in respect to age, which our author has observed. This and another patient are the only ones in whom he has witnessed adhesion of the omentum to the uterus, and in both there was incontrollable vomiting.

*Case* 5. On the 10th June, 1829, our author was requested to see a lady, æt. 42, who had borne twelve children, had been twice severely affected with syphilis, and had suffered from the unskilful use of instruments. In the preceding January, after a sudden mental emotion, she felt "as if machinery were turning round inside her" in the situation of the uterus. She was then seized with a leucorrhœal discharge, for which she used an astringent wash, which made her worse and deranged the bladder. The catamenia, which had been regular, stopped. In April she had a considerable hæmorrhage from the uterus, followed by constant watery discharge. On the 8th June she had another profuse uterine hæmorrhage. She had no pain whatever. On examination, the vagina was found filled by a tumour, of very irregular shape, as large as an orange, very firm, lobulated, totally insensible. The os uteri could not be detected, but most probably the tumour took its rise from it. The upper part of the vagina was immoveable, as if from consolidation with the surrounding parts. On the 16th, our author proceeded to remove the tumour by ligature, with a Niessen's canula, and the assistance of Dr. Benson. With much difficulty he succeeded in inclosing about two-thirds of the tumour. The patient suffered no pain, nor did any hæmorrhage follow. The ligature was tightened morning and evening till the 18th, when the canula and ligature came away, the noose inclosing a portion of the tumour about as large as a walnut. Several pieces came away with a tepid injection, and the same thing had occurred after every tightening of the ligature, attended, after the first day, with a very strong smell of putrefaction. On examination there appeared no possibility of re-applying the ligature, and on using the speculum, the tumour had a rough broken surface, as if from very unhealthy ulceration. On the 24th June, the patient complained, for the first time, of a soreness and burning in the lower part of the abdomen and pelvis, with some hæmorrhage. This was relieved by leeches, &c. On the 8th July, a good deal of the tumour was found to have been destroyed by ulceration, which had spread to the sides of the vagina. The discharge was more profuse and purulent. Œdema of the lower extremities came on—there was no accession of pain or disturbance of the bladder. On the 5th September prostration set in, and on the 8th she died.

*Sectio Cadaveris.* Intestines lying nearest to the brim of the pelvis united to the uterus and to each other by organized lymph—great omentum free. General consolidation of all the parts in the pelvis and softness of their textures—pelvic glands greatly enlarged, several of them of scirrhous structure. Rectum united to the posterior wall of the vagina and uterus, its parietes lacerable. Whole posterior surface of bladder covered internally by the peculiar brownish incrustation attending ulceration, which was seen in several patches, rendering the bladder excessively thin. Vagina ulcerated almost throughout, particularly at its uterine extremity. Here some of the bunches of tumours still remained unaltered; it was difficult to determine their origin from the vagina, or os uteri, or both. The cervix and os uteri were completely destroyed. Part of the body and fundus remained, their colour of a brownish inky darkness, their texture soft and shreddy.

Our author is in doubt of the cancerous nature of the preceding case, neither can he give a precise name and definition to the tumour.

*Case* 6. Ann Coffey, admitted into hospital in May, 1828. On the 18th June, she complained of wind passing from the rectum into the vagina. On introducing the finger into the vagina, these adhered to the shells of the grotts of which the gruel was made. Yet no breach was detected in the recto-vaginal septum and the stools were expelled by the anus; there was no evidence of ulceration in the vagina or os uteri. She died on the 26th. A few days before death she had considerable hæmorrhage from the vagina, and several clots of blood were mixed with fœcal matter. By mistake she was not examined.

"This was the only case under my own care, in which I had reason to think that the destructive ulceration commenced, in the first instance, towards the fundus uteri, which I have very little doubt occurred in this case, and union having previously taken place between the diseased organ and some fold of intestine, the latter became perforated, in consequence of which the substances already mentioned passed at once into the uterus, and escaped from the vagina.

This is rather a rare occurrence, of which, however, I saw one other instance, in an examination after death, in which an opening had been made by the ulceration, in the centre of the fundus, and into the ileum, which had become united to it."

*Case* 7. Jane Shannon, æt. 50, admitted May 1828. Pain in the back shooting through the pelvis—constant leucorrhœal discharge from the vagina, sometimes tinged with blood—occasionally slight hæmorrhage. On examination a firm ring or band was found surrounding the vagina, about an inch and half from its pubic end. It was as hard as cartilage, about half an inch broad, constricted the cavity of the vagina at the part to the diameter of about half an inch, and scarcely admitted the finger, which could just touch the os uteri; this was irregular, hard, and gaping. The patient would submit to no treatment, and left the hospital. On the 21st Sept. she was re-admitted. She had great pain in the loins, shooting through the pelvis; was exceedingly nervous; the appetite bad; the bowels confined. The circular band in the vagina was nearly as before, but the os uteri beyond was much less prominent, and felt soft and indistinct and ulcerated. On applying the speculum, the parts beyond the constricted portion were in a frightful state of ulceration spreading in every direction, and extending nearly an inch within the cervix; the os uteri almost destroyed. She could not retain her water long, and in the course of a few days she had complete incontinence of urine, followed by loss of power over the sphincter ani. For more than a week before death the vagina was the common outlet for the foul discharge of the uterus, the urine, and the fæces. On October 7th, when making an exertion, she suddenly complained of a piercing and burning pain in the pelvis, so intense that nothing could relieve it. On the evening of the next day she expired.

*Sectio Cadaveris.* Body not much emaciated. Kidneys healthy. Ureters greatly dilated and elongated. The right was in some parts three-fourths of an inch in diameter, and its pelvis greatly enlarged; it was ten or twelve inches long, and serpentine in its course. The left was not near so large. The portion of the bladder which had been destroyed, which was about the size of a halfpenny, had engaged and destroyed the termination of the left ureter, whilst on the right side the ulceration had not extended to the orifice of the ureter, the last half-inch of which was so obstructed by the pressure of the diseased mass that not a drop of urine would flow through it. At the back part of the uterus, and rather to the left side, there was a rupture in the peritoneum about an inch and half in length. The rupture was evidently the result of the ulceration which had eaten through the back part of the uterus, until nothing was left but the peritoneal covering, which gave way after an exertion of the patient. An aperture of about an inch in diameter was found between the bladder and vagina, and another of nearly the same size between the vagina and rectum.

This is the only case which our author has witnessed, of fatal perforation of the peritoneum produced by cancer uteri. He believes it is very rare. He has only seen the scirrhous band surrounding and contracting the vagina in another case besides the present.

*Case* 8. A patient, aged 45, applied to Dr. Montgomery. She stated that for three months previously she had been labouring under a burning sensation in the pelvis, with occasional lancinating and darting pains; she had had a considerable discharge, at first leucorrhœal and afterwards resembling pus; and she had experienced, at the same time, a difficulty in passing water, accompanied by a severe sensation of burning and scalding in the urethra. Two months previously she had applied to a medical man, who pronounced the case to be gonorrhœa, and prescribed cubebs, which she took for a month, and then, finding herself worse, gave it up. She had lately had one or two hæmorrhages, and the discharge had become discoloured and foul. On examination Dr. Montgomery found cancer uteri so fully established, that in four months she had fallen a victim to its ravages.

This case is calculated to inspire caution, and to impress upon medical men the necessity, the imperative necessity, of examination of the vagina and uterus in the discharges of females. We think that our readers will coincide with us in opinion, on the interesting character and practical value of the foregoing cases. Surgeons are not so well acquainted as they should be with the malignant diseases of the uterus, and that we have frequently observed. From the cases which we have witnessed, we should say that Dr. Montgomery's conclusions are, on the whole, very correct. We must find room for his concluding observations.

"If it appears that in the relation of the foregoing cases too little mention has been made of the practice adopted in the treatment of the disease, it should be recollected, that such was not the object of this paper, and even if it had been, I had nothing new to offer on the subject: every thing failed to cure, and the means of relieving pain in such cases are too well known to require even to be mentioned.

Upon the use of *Iodine*, however, as a remedy but lately sanctioned in practice, and by many supposed to possess great powers in cases such as I have been describing, I may observe, that as far as the result of ten of those cases in which I have used it, may warrant me in pronouncing upon its merits, I am of opinion that it will disappoint the expectations entertained of its powers in this disease; at least, it has done so completely, in my hands, in every case except that of Sarah Kearney, p. 427, where it certainly produced extraordinary, though temporary benefit in the condition of the patient, but had little or indeed no power in arresting the progress of the disease.

The use of the *Speculum uteri* also, may to some be a matter of novelty, as it certainly is, of interest. In these cases, and in many others, I have found it a most valuable assistant, by means of which, and in general with very little inconvenience to the patient, we can submit to the eye the state of parts, whose condition otherwise must necessarily be determined by little better than conjecture; let me however offer one observation, in the way of caution, regarding its use in these cases, where destructive ulceration *has been some time going on;* under such circumstances its introduction, however skilfully managed, may be productive of unpleasant consequences, by lacerating parts already almost eaten through by the disease; one instance of this occurred under my own observation, in which the vesico-vaginal septum, already deeply ulcerated, gave way upon the introduction of the speculum, and incontinence of urine, of course, immediately afterwards took place, and the additional misery thence ensuing was, very naturally, attributed by the patient to the operation, and not to the disease. It will not be out of place to mention here, that I have lately used this instrument in the adoption of a practice which, though now spoken of as new, was accurately described by Nigrisolius in 1665, I mean the application of leeches to the os and cervix uteri; this I have done three times in a case of irritable uterus, and with the most satisfactory effect.

This appears a fit place to allude to the operation of excision so much practised of late on the continent for the cure of cancer uteri; and certainly, as far as my experience has enabled me to judge, I feel quite prepared to declare my conviction *of its almost universal impracticability, and of its utter inutility when the disease really exists and is de-*

*veloped:* the first morbid changes in the organ usually take place at a time of life when disturbance in function is regarded as a matter of course, and uneasy sensations as a natural consequence, and even these are only occational, so that the evil creeps on unperceived, until a profuse hæmorrhage, an acute pain, or a foul discharge, first sounds the awful alarm to the patient; and when an examination is made, the disease is detected fully formed, and in general with consolidation of the neighbouring parts; the pelvic glands being in the great majority of cases contaminated by the disease almost from the beginning.

It is moreover quite ascertained, that the disease, instead of first shewing itself in the cervix, or os uteri, very frequently commences in some of the appendages of the uterus involving the surrounding tissues, or in the upper part of the organ, and thence spreading downwards, manifests itself last in the cervix; so that even should we succeed in removing the part which we have ascertained to be diseased, we leave *the disease* still behind. As a matter of observation on the subject, I may state, that not one ot the cases of the disease which I have met with, would have admitted of or justified any modification of this operation.

The dilatation of the ureters, which has been so frequently noticed in the above cases, is referred by Andral, Precis. tom. ii. p. 685, to the pressure of tumors on the bladder, by which its cavity is almost completely obliterated. This explanation is altogether at variance with my observations.

In the case of Clarke, for instance, where the cavity of the pelvis was more occupied by tumors than in any of the others, there was no distention of the ureters, because the disease had not encroached on their terminations in the bladder, while in every instance in which they had become involved in the morbid change the distention was found to exist, and sometimes considerably more at one side than the other, as the termination of the one happened to be more or less involved in the morbid thickening.—See p. 450. And I may add in addition, that within the last week I examined the body of a patient who died of this disease, and in whom perforation of the bladder had taken place: there was no tumor to press on the bladder, and while the right ureter was distended to the size of one's little finger, the left remained unaltered."

---

